# Spontaneous Cure of Organic Disease of the Heart

**Published:** 1880-03

**Authors:** David Wooster

**Affiliations:** San Francisco


### SPONTANEOUS CURE OF ORGANIC DISEASE OF THE HEART.

BY DAVID WOOSTER, M.D., OF SAN FRANCISCO.

*Case of Heart Clot—Death—Necropsy.*—The following case of spontaneous cure of very grave alterations of two sets of valves is furnished here as additional evidence of the declaration that—

In general, diseases of the heart do not necessarily tend towards a fatal termination, but on the contrary, towards recovery; or towards a condition in which the functions of the heart can be performed nearly as well as before the pathological change.

It has been long known that certain inflammations are specially liable to be followed by thrombi in the heart.

Among these, acute inflammation of the tonsils was observed to be a percursor of heart-thrombi, by Bouillaud, as long ago as 1838. His observations have since been occasionally verified at autopsies. Bouillaud lays it down as a law, that ante mortem thrombi are always found in one or more of the cavities of the heart in all cases of fatal pleuro-pneumonia. The case here reported, then, is not singular; but it is hoped that the narration and explanations may be found of practical value.

Miss M. M., the age of twenty, was teaching school in the winter of 1866–7, in Connecticut. She used to wade through deep snow to her school, and then remain on her feet all day in a cold room. Her feet and skirts were wet or damp almost every day, and she was rarely comfortably warm, except at home after school. She was seized with "internal rheumatism," from which she "nearly died;" and was confined to her bed for some weeks. After that, a doctor, in some western city, told her the sound of her heart was like that of "a boy drawing a stick along a picket fence. She has had since her rheumatism "shortness of breath" when hurrying, and especially in going up stairs. Some weeks ago she ascended four flights of stairs to her school room, and fainted away—falling down—but "came to" soon after with great pain and pressure about the heart. I gleaned these facts in conversations with her the last two days of her life, and from her friends since her death.

About three months ago she consulted me for what she called "rheumatism of the jaws." She could not separate her teeth the eighth of an inch, and she said it hurt her to swallow, as if her tonsils were sore. She continued teaching while taking remedies, and recovered. Again, on Monday, January 10th, Miss M. sent for me; this time sick in bed, with her teeth set, and in great pain at the right angle of lower jaw and in front of the ear. An attempt to open her mouth produced intense suffering. An anodyne having no effect, with a pine wedge she succeeded so far in opening her jaws, that on Tuesday I thought it safe to give her a thorough emetic of fluid extract of lobelia and ipecac. The relief was prompt and permanent, and the teeth would separate, by the will alone, about an inch. For the pain and febrile symptoms (the excretions being as they should be,) I gave her the following: Quin. sulph. gr. x, tr. opii. gtt. xl, acid muriatic, gtt. iij, at one dose, in a wine-glass of water. This dose was followed by rest, which she greatly needed, sleep and profuse perspiration. In twenty-four hours, that is, on Wednesday the 12th, this dose was repeated. In the mean time an abscess of the right tonsil had opened spontaneously, and she was spitting pus and mucus very freely. She said she felt perfectly well. Her pulse, respiration and temperature were about normal, except that her pulse had that "combed" quality indicating a previous rheumatism. Her appetite was good though sparingly indulged.

Thursday, in the morning, I laid my ear over the valves of the heart, and heard a rasping sound, but could not say whether it was friction or murmur, whether exo- or endo-cardial. She had no pain about the heart. I now heard for the first time of the rheumatism of twelve years ago, and that she had been teaching fourteen years. She now took alkaline remedies every four hours, and lavender and ammonia frequently. The alimentary way was clear, the renal organs acting sufficiently, but there was a slight brick-dust precipitate in the urine. She had spells of hacking cough, she said, to raise the secretion of the abscess, which trickled down her throat. At these times she would get purple in the face.

Friday, 9 A.M., better in all respects. Heart not examined, pulse not suggestive, except of an old rheumatism, and of slight nervousness. She said she was able to get up and dress, and asked permission to do so. She was advised to remain in bed at least another day.

At 6 P.M., on Friday, I received a telegram that she was dangerously worse, had had a sinking spell. Saw her at 8 P.M. There was no lividity of face or hands, ears, lips or nails; but the pulse had lost all its normal characteristics. It was no longer rythmic in any sense; no *vis a tergo;* no decided beat; an occasional pulse, possibly two or three in succession, of considerable force, then a thin flat line flowing under the finger; then an interval of stillness, more or less prolonged; then a few rapid faint taps, scarcely completed, without accent; and then rest, and a flowing, tiny streamlet, full of irregularities, intermissions and half hesitations, impossible to describe with entirely appreciable accuracy. The clanging of the heart is astonishing, but the patient says it is nothing new, but is of old date, and she described the sound, as before, by the simile of the stick drawn along a picket fence.

Hot fomentations and mustard to left chest; hot bottles to feet, as usual; Seidlitz powders, in broken doses, they being most grateful to her increasing thirst. She continues drinking a solution of this saline occasionally. The pulse cannot be counted, because of its irregularity, intermittence and flowing quality. The clang of the first sound, acccompanied by a palpable purring heard and felt all over the chest, is something frightful to observe, in a lady laughing and talking, and protesting she is in no pain, and that as to the body generally she feels almost well. Her tongue is only slightly coated, moist and not glutinous. She is not purple, and her face does not look puffy; perspires freely.

The signs, classic and clinical, of forming clots in the heart are undoubtedly well marked. The second sound of the heart is not extinct, nor attended with murmurs that can be detected through the noise; but it is altogether singular. It is audible at the usual side, between second and third cartilages, an inch to left of breast bone; but it is a faint tap, like that of a finger tapping feebly on a leather book-cover moistened. This sound, when heard, is immediately overwhelmed by the muffled loud, prolonged *chung* of the first sound. Often several second sounds are lost to the ear and then occur again, like the breath of a tap, they are so exceedingly feeble. This sound the autopsy has proved to proceed alone from the pulmonic semilunars, for the aortic values were agglutinated by their edges. Quinine, with elixir of bark; salines continued; brandy, besides the lavender, and ammonia, if needed.

Saturday, 10 A.M. Lividity, constantly varying from slight purple to leaden, the percursor of the end, is spread over her features and equally obvious in the extremities.

Pulse cannot be counted for any part of a minute. The blood occasionally runs through the radial, a thin narrow vascillating feeble stream; a pulse or two, and then a pause of alarming duration; then the same phenomenal pulse repeated. Respiration has increased in frequency to fifty times a minute. She now says, "It seems as if strings from all parts of the surface of the body, from the fingers and toes, centered in and tugged at the heart; that a string is around the center of the body, by which some one is trying to pull her down through the bed; that it seems as if one is standing on the ball of one foot, his whole weight on the chest over the heart. It is not pain, but pressure and anguish."

Saw her again at 1 P.M. Same symptoms. Four leeches over valves; bicarbonate of soda and bicarbonate of potash alternated hourly in twenty grain doses, largely diluted; champagne in regular doses, reinforced with brandy if the sense of sinking required.

7:37 to 9 P. M., Saturday; my last visit: All symtoms the same, except that pulse can be counted, and it is found that the heart is contracting only eighty times per minute. The pulse stopped often during the hour and a half I remained, and accurately corresponded to the observation of the morning visit. Excretions as they should be, and under control of the will; able to rise up to drink; says she could stand up, if she wished; is cheerful, but restless, and in great distress from the sense of pressure and constriction. One-fourth of a grain of acetate of morphine, to be repeated in four hours if needed.

At 5 A. M., Sunday morning, she expired by gradual asphyxia, conscious and able to talk up to three minutes before death, as I was assured by her very intelligent sister and mother. The morphine gave her great relief. She took the second dose.

Necropsy twelve hours after. The heart and lungs alone examined. Each pleural cavity contained at least a pint of serum of a faint straw color. No signs of pleuritis or pneumonia. No old adhesions of pleural surfaces. No solid spots in any part of the lungs. Lungs not engorged or edematous. The serum in the sac was doubtless transudative pure and simple, and was the result of an effort of nature to relieve the blood-pressure on the heart; an authoritative suggestion, if its cause be correctly stated, for copious venesection. The pericardium contained not less than four ounces of high-colored serum, which furnished a bright, pinkish, jelly-like, thin coagulum in the pan into which it was squeezed from the sponge. This is supposed to have been of very recent inflammatory origin. At the root of the heart the visceral pericardial layer showed equivocal signs of interstitial lymn-deposit, and the whole inner pericardial layer looked less pearly-lustrous than usual.

The right auricle was first opened. This auricle was much enlarged by dilatation, though its walls were of normal thickness. Its cavity, it was judged, would contain about six ounces of fluid, although its capacity was not measured. This auricle contained an ante-mortem clot of moderate dimensions, extending into the appendix auriculi, firmly adherent and closely intertwined among the musculi pectinati. It was pendulous from the appendix, and at each contraction of the auricle must have dropped into the tricuspid opening, and thus impeded the flow of blood into the right ventricle, and so suspended or interrupted the pulse.

The tricuspid was not appreciably altered.

The right ventricle was of normal size, and its walls of normal thickness. The pulmonic semilunar valves were supple, translucent, and not reddened either with inflammation or post-mortem imbibition. There was no clot in the pulmonary artery or between its valves.

There was a white resistent clot in the right ventricle, loosely restrained by the chords of the tricuspid, but the mass of the clot was susceptible of free motion. Scarcely a drachm of purple blood coagula in the right auricle and ventricle; no fluid blood, nor sign of endocardritis in either. The clot in the right ventricle must have floated towards or into the orifice of pulmonary artery at every systole of the ventricle, and so obstructed the passage of the blood into the lungs and towards the left side of the heart. This, with the clot in right auricle, would sufficiently account for the lividity of patient the last thirty-six hours of life, especially after coughing a few times.

The left auricle contained no white clots, was of normal size and aspect, and showed no inflammatory traces.

The left ventricle being opened, water was poured into it, and, the aorta being held, the water passed through the mitral into left auricle. The edges of the two mitral folds were united from the right towards the left, to the extent of more than one-half the length of their free margins. This was an old firm adhesion or rather fusion, and the mitral, in place of consisting of two leaves, consisted of one dense three-times-thickened tendinous disc, perforated in its left circumference with an aperture into which could be pushed the tip of a small little finger. On the auricular surface of this disc was a sulcus half the depth of the thickness of the mitral disc, showing the line of fusion of the original leaves. The length of this sulcus or cicatrice-mark was by measurement five-eights of an inch. The length of the opening in the disc, where its sides were brought together, was slightly less than half an inch. These measurements were made on the auricular face of the disc.

It is seen, of course, that the affection at the mitral is obstructive to five-ninths the capacity of the orifice, and patent (incompetent) to four-ninths, of which opening, perhaps, one-fourth was permanent.

The suggestions of this pathological condition are curious and eminently instructive. I will first premise that the left venticle was normal in size, in capacity of cavity, in thickness of walls, and in freedom from endo-cardial signs, although its tissue was very friable, and had possibly undergone fatty substitution to some extent. On this point no examination was made. The point I wish to present, as to the mitral, is, that it was five-ninths competent by the fusion of its edges, and that the competency was in the part of the valve where it would do most good, namely, in that portion (towards the base of the heart) where most blood would impinge upon it. The incompetency, on the other hand, was where it would do least harm in permitting regurgitation, namely, towards the apex of the cone; and where it would most nearly serve its normal purpose of permitting arterial blood to flow through [it from the left auricle, for it corresponded in place to the mouth of the auricular conoid, being directly opposite the appendix auriculi. And futhermore, such was the location of the opening in the disc, that when the tendinous ring of the mitral descended towards the rising apex in systole (the very moment that regurgitation must take place if at all), the inner aspect of the left part of the left ventricle would be applied so as to partly occlude this hiatus of incompetency. In consequence of this reparative triumph of nature, accomplished nearly twelve years ago, the woman suffered only a minimum amount from mitral regurgitation.

The aortic semilunars were in a still more anomalous condition. They were three times thickened, and fused each to the other, beginning at their acute angles of origin from the aortic wall, to such an extent along each curved margin that there resulted a disc as in the mitral, with an aperture in the centre, likewise accurately measuring six lines, when its edges were brought together; while the segment of the aorta itself, cut off close to the roots of the valves, measured inside, when it was flattened, fifteen lines (1¼ inch).

Hence it will be seen that the incompetency of the aortic valves, even at most, was only six-fifteenths of the aortic lumen, or perhaps almost exactly the same as that of the mitral disc. But on the other hand, the obstruction to the outward current of the blood into the aorta was by the same reasoning nearly nine-fifteenths of the normal lumen. This obstruction, it will be seen, was compensated by a corresponding regurgitation through the permanent hiatus of the same diameter in the mitral. And the left auricle was saved from hypertrophy and dilatation by the dilatation of the right auricle, which acted as a safety chamber and reservoir for the other three cavities.

But to continue with the conditions of the aortic semilunars. They no longer exhibited more than a vestige of their normal shape and aspect. They were thick, opaque and tendinous; fused into one hollow pyramid open at the apex, so that only the point of a small little finger could be pushed through it; or to be more accurate, a cylinder just five lines in diameter could barely be crowded through the hiatus in the centre of the fused semilunars. (The circumference of a cylinder that could be pushed through the hiatus of the mitral disc is accurately seventeen lines, or, diameter five and two-thirds lines, instead of five, as that through the aortic semilunar disc.) But as in the mitral this hiatus was so arranged that it allowed vastly less regurgitation than exit. The anterior semilunar is fused by a thin line along its middle, from its attached to within a little less than three lines of its free edge at the corpus arantii of that valve; and this strip, of less than three lines, is supple and drops easily towards the axis of the artery. The other two segments are so repaired as to partially overlap, by their free edges, the free margin of this one, so that when water is poured into the artery it falls down behind these (for the most part) rigid valves into the aortic sacs, and rising presses the sides of the pyramid towards the axis of the orifice of the artery. Rising still higher, it shuts the free edges of the two valves down over the other, the margin of which becomes tense by the filling of the root of the artery, and then there remains a hiatus through which a rod only one line in diameter will pass.

Now, my intelligent readers will perceive that here is an almost exact balance—primary pathological change, and secondary pathological compensation.

We will suppose, for the occasion, that the primary rheumatic affection touched first the aortic semilunar segments, and in a few days caused the thickening and partial fusion of these segments. This would have obstructed the systole movement of the blood, and the tendency would have been towards hypertrophy of the left ventricle ; but the disease immediately after affects also the mitral, and its valves losing a portion of their elasticity and resisting tone, permit regurgitation to the extent of the obstruction at the aortic orifice.

Now here we have, first, aortic obstruction; second, mitral regurgitation-compensation. Now the rheumatism, or what is the same, the endocarditis, abates, but leaves permanent interstitial deposits in the semilunars, and leaves their margins fused and rigid, so that they are no longer competent. This, without compensation, is a terrible lesson, suggesting the gravest consequences. But nature comes to the rescue, like the ever-provident mother she always is, and inflicts another lesion by way of compensation. The mitral segments are also thickened and fused, and hardened to the same relative degree; and less blood is permitted to pass from the left auricle into the left ventricle, by reason of the nearly half obliteration of the mitral orifice.

Here we have clearly before our eyes, mitral regurgitation compensating aortic obstruction; and mitral constriction (obstruction) compensating aortic regurgitation (incompetence).

If the disease stops at this point the patient is practically well, though the pathological charges remain during life. But if these two orifices, the mitral and aortic, through which all the newly oxygenated blood of the body must pass in ceaseless and rapidly-repeating circuit, are permanently constrict to the extent of nearly one-half, what will become of the obstructed volume of the blood? Obvious it must have some resting place until it can pass on its destined course.

Would not this induce permanent, gradually increasing congestion of all parts of the body? Theoretically it would, but practically this is not the result. Instead of this fatal condition, nature, left to herself, or judiciously aided, performs another miracle. She enlarges the right auricle to serve as a temporary expedient in case of excessive flow of blood towards the heart. In this elastic chamber it can accumulate until the pressing flood begins to ebb again into the thousand chambers ready to receive it.

But the great feat accomplished is the addition of defective appetite to the "heart disease." By this paradox less food is desired by the patient, at the same time that less is required by less by the body itself. The blood diminishes in quantity to the capacity of the central organ, the heart, for its reception and transmission. Here again our patient arrives at health; health of a lower standard, it is true, but still unconsciousness of any lesion except the lack of the strength or endurance she thinks she ought to possess. In this condition, if the strength or endurance be not overtaxed, there is reason to believe a patient might live not only twelve years in unsuspected health, but even fifty or sixty.

*Corrollary.*—Very great alterations in the organic condition of the heart are not of fatal tendency.

